# Face recognition and memory in congenital amusia

**DOI:** 10.1371/journal.pone.0225519

**Published:** 2019-12-02

**Authors:** Weidong Tao, Huayan Huang, Hanna Haponenko, Hong-jin Sun

**Affiliations:** 1 Department of Psychology, School of Teacher Education, Huzhou Normal University, Huzhou, Zhejiang, China; 2 Department of Psychology, School of Education, Lingnan Normal University, Zhanjiang, Guangdong, China; 3 Department of Psychology, Neuroscience and Behaviour, McMaster University, Hamiliton, Ontario, Canada; Universiteit van Amsterdam, NETHERLANDS

## Abstract

Congenital amusia, commonly known as tone deafness, is a lifelong impairment of music perception and production. It remains a question of debate whether the impairments in musical domain observed in congenital amusia are paralleled in other non-musical perceptual abilities. Using behavioral measures in two experiments, the current study explored face perception and memory in congenital amusics. Both congenital amusics and matched controls performed a face perception task (Experiment 1) and an old/novel object memory task (for both faces and houses, Experiment 2). The results showed that the congenital amusic group had significantly slower reaction times than that in matched control group when identifying whether two faces presented together were the same or different. For different face-pairs, the deficit was greater for upright faces compared with inverted faces. For object memory task, the congenital amusic group also showed worse memory performance than the control group. The results of the present study suggest that the impairment attributed to congenital amusia is not only limited to music, but also extends to visual perception and visual memory domain.

## Introduction

Congenital amusia is characterized as a lifelong musical impairment in pitch discrimination and recognition [1], or in beat synchronization [2]. Congenital amusia is estimated to affect about 4% of the general population in the United Kingdom [3] and 3.4% of the population in China [4]. The most recent report of prevalence of congenital amusia is 1.5% [5]. It is generally believed that the musical impairment that characterizes congenital amusia cannot be explained by prior brain damage, hearing impairment, level of education, lack of normal musical exposure or general cognitive deficits [6,7]. Previous studies showed that people with amusia have impaired recognition of fine-grained pitch changes in melodies, such as when another person sings out-of-tune [8]; they also have deficiencies in experiencing emotional prosody [9], although some of them still sensitive to musical emotion [10]. Amusics are also impaired in musical memory, singing ability, and tapping along to a beat [11].

An important question underlying congenital amusia is whether the observed impairment is restricted to musical domains. Studies have found that congenital amusics are often impaired in the recognition of fine-grained pitch changes but not in other non-musical auditory stimuli [6, 12, 13], such as human voices or environmental sounds [14]. Other studies, however, have put forward the possibility that congenital amusics’ deficits may extend to non-verbal sound attributes like timbre [15,16]. Amusia can also show deficits in the processing and acquisition of tonal and atonal language in early human life [4]. This suggests that individuals with congenital amusia also experience pitch deficits with language stimuli [4, 16–21]. Thus, music processing and language processing might share similar cognitive mechanisms [9, 22, 23].

Associations between pitch processing and visuospatial processing are controversial. A study by Douglas and Bilkey (2007) examined spatial abilities in congenital amusics and found that deficiencies went beyond the musical domain [24]. Specifically, they demonstrated that people with congenital amusia performed worse than controls on a mental rotation task. Along the same line, our recent behavioral studies showed that, compared with controls, people with congenital amusia perform worse on spatial representation and mental rotation task [25]. Similar studies also suggested that pitch processing may rely on cognitive mechanisms that are similar to those used for spatial processing [26,27], although, some other researchers failed to find an association between congenital amusia and deficits in spatial processing [28,29]. To date, investigations into whether congenital amusics have other kinds of non-musical deficiencies have been limited to the examination of language ability and spatial ability.

Recently, several studies explored the link between amusia and face recognition deficit [30–31]. For example, Paquette et al (2018) found that some tone-deafness cases also had deficit on face recognition [30]. A study found that 25% developmental prosopagnosia were impaired in fine pitch discrimination [31]. About 30% of congential amusia was diagnosed with dyslexia [32]. These studies showed that deficit might not be limited to one modality.

It seems to be reasonable to speculate that congenital amusia could have deficiency in face processing due to the similarities in underlying processes involved in both face and melodic processing. An infant study by Trehub (2001) suggested that melodic processing is processed in a holistic manner [33]. For instance, dividing well-known melodies into small segments before scrambling them impaired listeners’ recognition performance significantly. This showed that the systematic arrangement of melodic elements is essential for the recognition [34]. In particular, a previous study demonstrated that meter might be a complex acoustic-holistic process which requires processing of sound intensity and rhythmical periodicity, while rhythm processing requires combining relationships between a series of durations into a whole [35]. With respect to face processing, a considerable number of studies have suggested that holistic grouping is also essential [36–41]. Therefore it seems that both faces and melodies are processed holistically. Thus, musical processing may very well share similar cognitive mechanisms with face processing.

The studies of neural mechanisms of congenital amusia have shown that amusia may be a connectivity disorder between an intact auditory perceptual system and the frontal cortex [42]. Stewart (2011) proposed a simplified model of melodic processing which have three processing stages, that means the impairments could took place at any or all of these processing stage [43]. For instance, this problem in connectivity shares similarity with the neural mechanism of congenital prosopagnosia (failures to develop normal face recognition) [44]. Both deficits represent altered connectivity between an intact core perceptual system and the frontal cortices [45]. Both music and face processing are complex cognitive tasks that might engage distributed networks, linking together distant cortical regions. When the connectivity fails, selective cognitive impairments arise. Since a music and face processing share similar neural mechanisms, it should be worthwhile to examine whether people with congenital amusia would have a deficit in face perception.

In addition to perception, memory deficiencies have also been demonstrated in amusics. Findings of several studies on deficiencies in congenital amusics demonstrated brain’s potential to possess pitch-specific memory [46–48] along with timbre memory [15]. The memory tasks in these studies all used auditory stimuli and required participants to complete a recognition task by judging whether a pair of stimuli were identical or different based on certain auditory characteristics (pitch, tone, timbre, or verbal material). Similar to perceptual deficit, it would be important to determine whether the potential memory deficits identified in amusics represent general memory deficits independent of modality of the sensory input. To our knowledge, little research has been undertaken to explore whether this disorder is also accompanied by memory impairments outside of music domain.

The current study investigated whether people with congenital amusia exhibited deficits in face perception and face memory compared with matched controls. Such a study potentially provides new evidence as to whether congenital amusia is a domain-specific deficit. Thus, two experiments were performed to test for performance differences in visual stimuli processing between congenital amusics and matched controls. In Experiment 1, both congenital amusic participants and matched control participants were required to make a “same” or “different” judgment in a face perception task before they completed the Montreal Battery Evaluation of Amusia (MBEA). Both upright faces and inverted faces were used as stimuli. The inverted face stimuli served as control stimuli which contained the same low-level visual components. While discrimination of upright faces would require the expertise of face processing and discrimination of inverted face would demonstrate the processing of low-level visual information. In Experiment 2, a new group of amusic participants and matched controls were required to complete a recognition task (indicating old/new) for newly learned materials (pictures of faces and pictures of houses) after they completed the first three subscales of MBEA. House pictures served as control stimuli in order to assess whether the deficit (if any) would be specific to face stimuli.

## Experiment 1

### Materials and methods

#### Participants

Fifty-four potentially congenital amusic participants who self-reported that they feared to sing in public, sing out of tune, and had difficulty keeping rhythm when singing or dancing, were recruited from Zhanjiang Normal University. All participants were assessed individually via the Montreal Battery Evaluation of Amusia (MBEA) [49]. Individuals with scores of less than 65 on the first three subtests (i.e. scale, contour, and interval subscales) were considered true congenital amusics [19]. Using these criteria, 14 congenital amusic participants were recruited from a sample of 54 self-reported amusics, formed a sample of 1 male and 13 females, with an average age of 19.9 years. Meanwhile, another 14 participants who self-reported that they were good at singing were recruited to participate in this experiment and served as the control group. Control participants (1 male and 13 females with an average age of 19.7 years.) were matched to the amusic group according to age (t(26) = 0.784,p>0.05), sex (each group have 1 male and 13 females), and years of education (all participants are undergraduate students). All participants had a normal or corrected-to-normal vision and self-reported right hand dominance.This study has been approved by the IRB School of Teacher Education at Huzhou Normal University. All participants gave written informed consent and were paid 20 Yuan for their participation.

#### Stimuli

The Montreal Battery of Evaluation of Amusia and face perception tasks were compiled using E-Prime 1.1 (Psychology Software Tools, Pittsburgh, Pennsylvania, USA). For the face perception task, 30 neutral greyscale faces of Chinese men were selected from a standard Chinese face database developed by Bai, Ma, Huang, & Luo (2005)[50]. The stimuli were displayed on a 17-inch SVGA monitor with a resolution of 1024 × 768 pixels, and each face was displayed in 160 × 240 pixels, resulting in a visual angle of 2.05º × 3.08º at a viewing distance of 75 cm. Faces were simultaneously presented in pairs side by side and the participants were required to make a “same” or “different” judgment. The face perception task followed a 2 (Group: amusic group, control) × 2 (Match: matched pair and non-matched pair) × 2 (Orientation: upright face, inverted face) design. Both self-reported amusic participants and matched controls completed the face perception task before completing the MBEA (30items in each subscale) where both accuracy and reaction time were recorded.

#### Procedure

For face perception task, each trial began with a black central fixation point comprised of a “+” symbol that was shown for 1 second. Following this, two faces were presented on the screen at the same time, one on the left and one on the right. The faces remained on the screen until the participant made a “same” or “different” response as quickly and as accurately as possible by pressing the “1” key (with the right hand index-finger) when the two faces were identical and pressing the “2” key (with the right hand middle-finger) when the two faces were different. After that, the text “Waiting for the Next Trial” was presented for 1 second. The reaction time and the error rate were recorded. For each participant, the practice trial consisted of 20 face pairs and the formal experiment consisted of 80 face pairs. Both the amusic and control groups completed the subtests of the MBEA that measured scale, contour, interval, rhythm, meter, and memory.

### Results

Trials with RTs larger than two standard deviations from each group’s mean or less than 200 ms (4.55% of trials for the congenital amusic group, 5.17% of trials for matched controls) were excluded from the analysis. Only the mean RTs for correct response trials were analyzed. There was no significant correlation between RT and error rate (r  =  0.012, p  =  0.861), suggesting that there was no speed/accuracy trade-off.

Accuracy scores for each subtest of the MBEA (scale, contour, interval, rhythm, meter, memory) were significantly lower for the amusic group compared with the control group: (t(26)  =  -18.78, p < 0.001, Cohen’s d = -1.76; t(26)  = -8.079, p < 0.001, Cohen’s d = -3.15; t(26)  =   -10.626, p < .001, Cohen’s d = -4.11; t(26)  =   -2.939, p  =  0.007, Cohen’s d = -1.20; t(26)  =   -2.154, p  =  0.040, Cohen’s d = -0.86; t(26)  =   -5.625, p < 0.001, Cohen’s d = -2.56). In addition, the average RTs on the subtests of scale, contour, and interval for amusia were significantly slower for the amusic group than for the control group (t(26)  =  2.561, p  =  0.017, Cohen’s d = 0.97; t(26)  =  2.454, p  =  0.021, Cohen’s d = 0.93; t (26)  =  2.583, p  =  0.016, Cohen’s d = 0.98). There were no significant differences between the groups’ reaction times in the subtests of rhythm, meter, and memory: (t(26)  =  1.981, p  =  0.058; t(26)  =  1.326, p  =  0.196; t(26)  =  1.975, p  =  0.059). The MBEA reaction times and scores for the amusic and control groups are shown in Table 1.

**Table 1:** Scores and Reaction Times (ms) in the amusia and control groups tested using the montreal battery of evaluation of amusia in Experiment 1.

	Group	Reaction time, mean (SD)	Accuracy, mean (SD)
Scale	Amusia	823 (326)	16.86 (1.70)
	Controls	557 (209)	22.82 (4.52)
Contour	Amusia	843 (441)	20.43 (3.11)
	Controls	527 (191)	28.07 (1.44)
Interval	Amusia	1013 (614)	16.71 (3.12)
	Controls	553 (258)	27.35 (1.91)
Rhythm	Amusia	951 (715)	22.07 (4.71)
	Controls	549 (256)	27.21 (3.81)
Meter	Amusia	1010 (590)	20.64 (4.49)
	Controls	766 (358)	24.79 (5.16)
Memory	Amusia	816 (382)	22.93 (3.34)
	Controls	546 (340)	28.57 (1.16)

**Fig 1:**
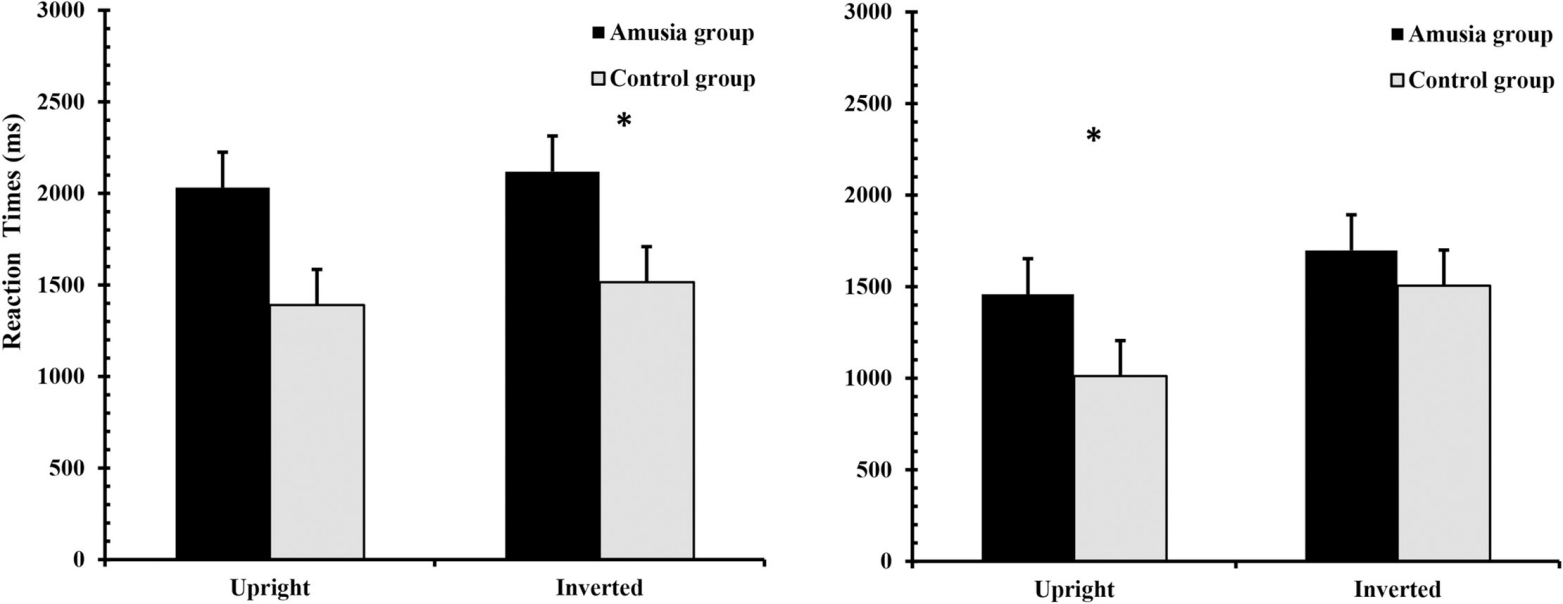
RTs for the amusia group and the control group in the face recognition task. Matched pairs (left) and non-matched pairs (right). Error bars represent the standard error of mean. Asterisks indicate statistically significant differences.

The pattern of results for RT can be found in Fig 1. RTs were analyzed using a 2 × 2 × 2 mixed-effects ANOVA that was conducted with Group (amusics, normal) × Match (matched pair, non-matched pair) × Orientation (upright face, inverted face). The main effect of group was significant (F(1,26)  = 6.22, p  =  0.019,*η*^2^ = 0.193): the congenital amusic group (M  =  1827 ms., SD  =  133.61) had slower RTs than the control group (M  =  1356 ms, SD  =  131.21). Furthermore, there was also a significant main effect of orientation (F(1, 26)  =  13.92, p  =  0.001,*η*^2^ = 0.349): upright faces (M  =  1473 ms, SD  =  89.03) elicited faster RTs than inverted faces (M  =  1709 ms, SD  =  109.20). The main effect of match was also significant (F(1, 26)  =  13.536, *p* = 0.001, *η*^2^ = 0.342). The RTs were slower for matched pairs (M  =  1763 ms, SD  = 128) than non-matched pairs (M  =  1418 ms, SD  =  75).

Only the interaction between match and orientation was significant (F(1,26)  =  7.137, p  =  0.013, *η*^2^ = 0.215). A simple main effect analysis indicated that there was a significant difference between upright faces and inverted faces for matched pairs (F(1,26)  =  12.25, p < 0.001, *η*^2^ = 0.28). The difference between upright faces and inverted faces was not significant for non-matched pairs (F(1,26)  =  2.06, p > 0.05). None of the other two-way or three-way interactions were significant (all p > 0.05).

Error rates were analyzed using a 2 × 2 × 2 mixed-effects ANOVA. The results showed that there was no significant main effect of group (F(1,26)  =  0.111, p > 0.05, *η*^2^ = 0.062) or match (F(1,26)  =  0.002, p > 0.05, η2 = 0.05). The main effect of orientation was significant (F(1,26)  =  7.749, p  =  0.01, *η*^2^ = 0.764), showing that upright faces (*M*  =  0.043, SD  =  0.013) yielded fewer errors than inverted faces (M  =  0.084 , SD  =  0.011). For interactions, only the interaction between match and orientation was significant (F(1, 26)  =  20.97, p < 0.001, *η*^2^ = 0.993). A simple effect analysis showed that there was no significant difference between upright faces and inverted faces for matched pairs (*F*(1,26)  =  1.45, p > 0.05, *η*^2^ = 0.210). However, there was a significant difference between upright faces and inverted faces for non-matched pairs (*F*(1,26)  =  24.10, p < 0.001, *η*^2^ = 0.996).

### Discussion

In Experiment 1, the error rate did not show a significant difference between the amusic and control groups, but this was most likely due to very low error rates found in both groups. More interesting results came from reaction times which showed that, for face perception, people with congenital amusia were slower compared to the matched controls. One reason for the slower face perception in amusics could be that the impairment extends to non-musical stimuli.

A more interesting possibility could be that this deficit represents a specific deficit for complex and over-learned stimuli like face. Human faces share some basic features, composed of eyes, noses, and mouths whose perception has been found to be mainly dependent on holistic information or configurational representation [38–41]. Music is an acoustic signal that requires people to segment, interpret and anticipate elements that unfold over time. Due to the nature of the information processing, face and music processing might share a similar mechanism for holistic processing. Therefore, it is reasonable to speculate that the amusic group would show some deficit for face processing. Recently a study explored the coexistence between tone-deafness and prosopagnosia and supported this speculation [30].

If slower perception in the amusic group represents a deficit in holistic processing, the deficit should be greater for upright faces compared with inverted faces. When processing upright faces, normal controls might use holistic information while amusic participants might use feature-based processing. Holistic processing is believed to be faster than processing feature-based information [36]. However, for inverted faces, it has been suggested the processing is achieved through feature consolidation [51]. Therefore, for inverted faces, one would not expect much difference between the amusic and control groups. Our data showed a clear trend for the discrepancy in group differences for upright vs inverted faces, but the group difference was more pronounced in non-matched faces (see right panel of Fig 1). For non-matched faces, our results showed a greater difference between amusic and control group in upright vs inverted faces. There was a lack of statistically significant difference for the interaction between group and orientation when we ran an ANOVA with all the data included. However, when we conducted a separate ANOVA for nonmatched face condition only (corresponding to the data in the right side panel of Fig 1), the interaction between group and orientation was significant (F(1,26) = 4.86, p<0.05). Non-matched faces might offer a more sensitive test as the reaction time in that condition might contain less noise as participants could produce a response as soon as they identified any feature differences. For matched faces, however, participants had to compare all of the features between two faces before they could reach a decision.

Although for non-matching faces, there was a group difference between upright and inverted faces, suggesting that the deficit in amusia was in the holistic processing of faces, no such pattern was seen in matched faces, in which the amusic group showed a deficit regardless of the face orientation. This suggests a general perceptual deficit in amusics. The fact that a comparable magnitude of deficit was seen in both upright and inverted faces suggests that this was not due to any lack of statistical power in the experiment. Rather, there was a robust group difference regardless of the orientation.

Given the deficit in amusia at the perceptual level, it would be necessary to examine other possible deficiencies in non-music domains. A second question related to the generalization of congenital amusics’ deficits is whether the deficits extend to face memory. Experiment 2 explored whether a difference between congenital amusics and normal controls exists in face memory.

## Experiment 2

### Materials and methods

#### Participants

Another 14 new amusics (12 females, 2 males ages 18–22, with average age of 20.1) and 14 new matched controls (12 females, 2 males, ages 18–22, with average age of 19.5, took part in Experiment 2. All participants had normal or corrected-to-normal vision. Both amusics and controls were undergraduates without formal musical training and none of them had any previous neurological or psychiatric history. This study has been approved by the IRB School of Teacher Education at Huzhou Normal University.All participants were self-reported right hand dominant. Informed written consent was obtained from all participants, and each participant received 20 Yuan RMB for his or her participation.

All participants were assessed with the first three subtests of the MBEA [50]. Participants whose scores were lower than 65 for the first three subtests (scale, contour, and interval) were considered as congenital amusics [22] and the amusic participants scored significantly below the matched control participants in these three subtests (t(26)  = -11.40, p < 0.001, Cohen’s d = -4.32; t(26)  = -9.26, p < 0.001, Cohen’s d = -3.49; t(26)  =   -10.42, p < 0.001, Cohen’s d = -3.94) (see Table 2).

**Table 2:** Scores in the amusia and control groups tested using the montreal battery of evaluation of amusia Experiment 2.

	Group	Accuracy, mean (SD)
Scale	Amusia	18.93 (2.27)
	Controls	27.29 (1.54)
Contour	Amusia	20.78 (2.33)
	Controls	27.57 (1.45)
Interval	Amusia	18.86 (2.91)
	Controls	27.50 (1.09)

#### Stimuli

The experiment comprised of a learning phase and a subsequent test phase. For the learning phase, 20 faces (10 male and 10 female) were selected from the standard Chinese faces database [50], and 20 houses (10 houses and 10 apartments) were selected from the Park Aging Mind Laboratory (http://agingmind.utdallas.edu/other-stimulus/). In the test phase, besides these old 20 faces and 20 houses, another 20 novel faces and 20 novel houses were selected. All the stimuli were displayed on a 19-inch SVGA monitor with a resolution of 1024 × 768 pixels at a viewing distance of 75 cm. Each face was 260 × 300 pixels (visual angle 4.07º × 3.51º) and each house was 320 × 240 (visual angle 5.00º × 2.81º).

#### Procedure

In the learning phase, the participants were presented with 20 faces and 20 houses in randomized order. Before the learning phase began, participants were informed that they would be tested on their memory of the studied faces and houses following the learning phase. Each trial began with a black central fixation “+” symbol, shown for 1 sec., and was then followed by a picture (a face or house) displayed for 2 sec. After 3 minutes break following the learning phase, participants performed an old/new judgement. Participant made a response by pressing the “1” key (with the dominant right hand index finger) if the picture had been previously presented in the study phase (the “old” condition) or pressing the “2” key (with the dominant right hand middle finger) if the picture was novel (the new” condition). The experiment displayed 40 pictures in the learning phase and 80 pictures (40 old and 40 new) in the test phase. All the participants were instructed to respond as accurately as possible.

### Results

The “old” and “new” responses were collected and combined into the sensitivity index of *d-prime* (hit rates were the correct answers to the novel responses; false alarm rates were the wrong answers to the old responses). Hit and false alarm rates of 0 or 1 were adjusted to eliminate infinite z values. Large *d-prime* values indicate greater discriminability.

**Fig 2:**
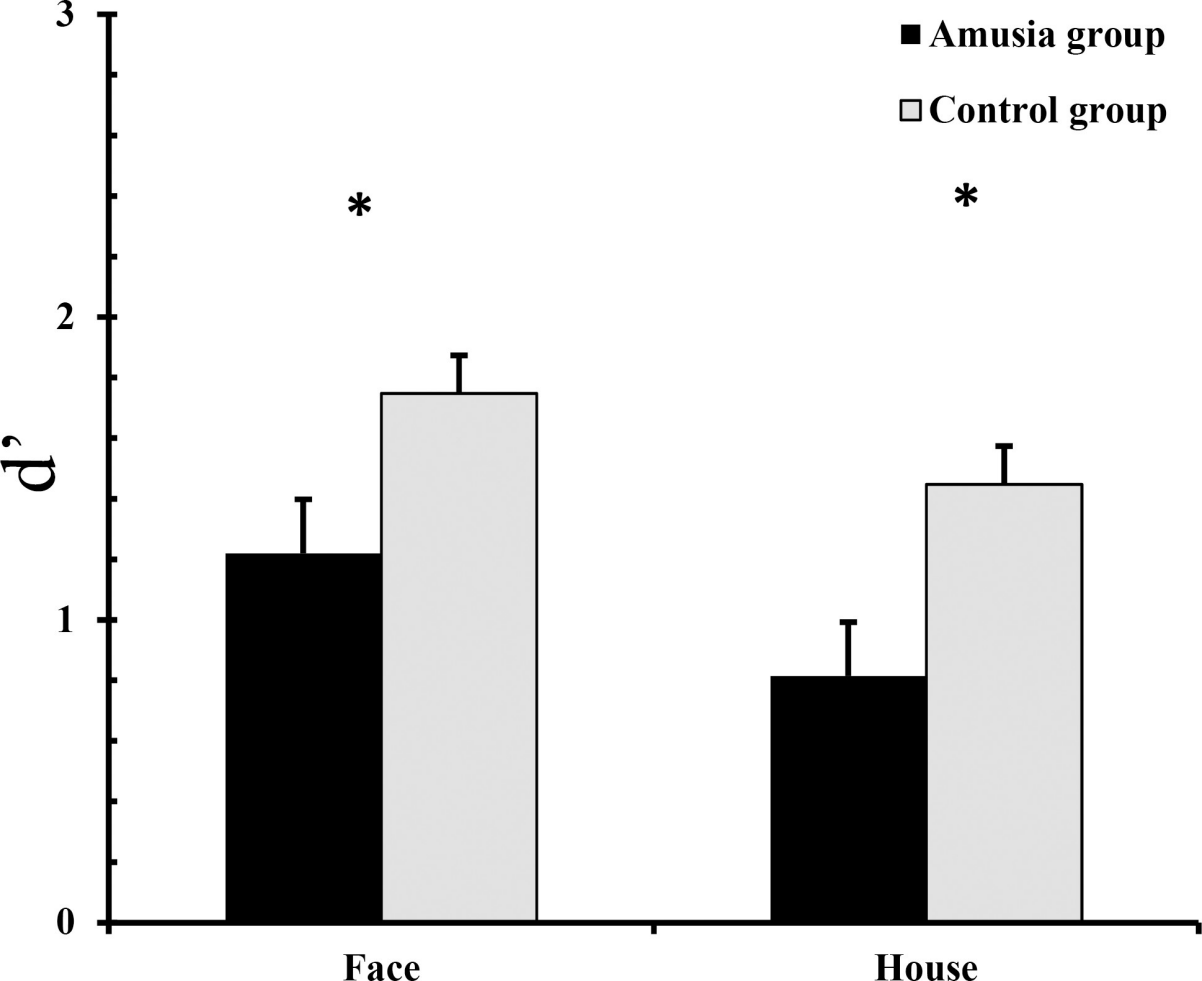
*d-prime* for the amusia group and the control group in the face recognition and house recognition tasks. Error bars represent the standard error of mean. Asterisks indicate statistically significant differences.

A mixed-model two-way repeated measure ANOVA of *d-prime* with group (congenital amusics or controls) as a between-subjects factor and object type (face or house) as a within-subjects factor indicated a main effect of group (F(1,26)  =  10.13, p  =  0.004, *η*^2^ = 0.28), with lower *d-prime* for the congenital amusic groups (M  =  1.02, SD  =  0.13) compared with controls (M  =  1.60, SD  =  0.13) (see Fig 2), as well as a main effect of object type (F(1,26)  =  8.69, p  =  0.007, *η*^2^ = 0.25), with lower d-prime for houses (M  =  1.13, SD  =  0.13) compared to faces (M  =  1.48, SD  =  0.09) (see Fig 2).

### Discussion

The present experiment investigated face memory of a congenital amusic group and a matched control group. The results showed that object discriminability (for both faces and houses) was lower among the amusic participants compared with the matched controls. This supports the hypothesis that the deficiencies of congenital amusics extend to other non-musical domains, including visual memory of faces and house.

The results also demonstrated that, for both congenital amusics and matched controls, the performance for recognizing faces was better than that for recognizing houses, suggesting better memory for faces than houses. Prior reports have provided evidence that faces are distinct from other objects and that configurational information is critical in recognizing faces [51,52]. Additionally, face perception is a fundamental skill for daily communication in human society, and most people are remarkably proficient when recognizing faces [53–55].

## General discussion

For both upright and inverted faces in matched pairs, reaction times for the amusic group were significantly slower than those for the matched controls. In addition, people with congenital amusia had lower performance than the control group for both face and house memory. These findings showed that the amusic participants not only had deficiencies in their musical ability (accuracy and reaction times on the MBEA), but also extend to the visual domain (face and house). There exists the possibility that the impairment of congenital amusia is not only exhibited in the musical domain but also is extended to the non-musical domains of face and object processing.

Furthermore, the findings from non-matched faces in Experiment 1 are in line with the possibility that music processing and face processing might share a common neural mechanism. Similarly, voice, which has been called the “auditory face” also has an inversion effect in an inverted audio paradigm, where participants have a more difficult time recognizing speech whose frequency or verbal dialogue was inverted. These findings are analogous to those of the classical face inversion effect, where individuals have a more difficult time processing and recognizing inverted over upright faces [56].

While previous studies showed that impairments of congenital amusia was limited to music domain [6, 12, 13, 43], our results were more in line with the hypothesis of cross-domain deficits. Such shared deficits across several neurodevelopmental disorders were reported. Several tone-deafness cases also had the disorder of face recognition [30] and 25% developmental prosopagnosia were impaired in fine pitch discrimination [31]. These researches showed that deficit in one modality was not necessarily limited to that modality. A four processing stages of melodic processing model was proposed by Stewart (2011), each processing stage were related to difference brain cortices, such as primary auditory cortex, secondary auditory cortex, inferior frontal gyrus. Inferior frontal gyrus in particular might be heavily involved in processing other modality information [43]. The neurobiology of amusia showed that the connectivity between the right inferior frontal gyrus and the superior temporal gyrus are reduced [57].

There is evidence that people who listen to music activate a complex bilateral network of temporal, frontal, parietal, and subcortical brain areas, with the activation of the temporal lobes being highly involved in the perception of music [58–60]. The neuropsychological and neuroimaging literature has highlighted the fact that face processing produces additional activation of the temporal regions [61,62], while studies of brain injuries have provided information about cases of impaired face processing, usually following bilateral occipito-temporal damage [38]. These findings suggest the possibility of an overlapping neural mechanism for face and music processing that is associated with activity in the temporal regions.

Another study also found that object recognition tasks elicited activation primarily in the left occipito-temporal region [63]. However, for processing of man-made objects, such as tools, activation was also found in the left posterior middle temporal cortex [61]. Neuroimaging studies have also consistently indicated that object processing involves the lateral occipital complex (LOC) [64,65]. From these studies, a hypothesis could be proposed that music processing and object processing share a similar mechanism involving the temporal cortex. In the current study, the amusics had worse performance than the controls on the face memory task. A possible explanation, which would need to be assessed in detail in future studies, could be that the deficits associated with congenital amusia may be reflected both in music processing and in face memory. Neuroimaging studies revealing differential activations in the temporal lobes of amusic participants compared with controls across different musical and nonmusical recognition tasks could also be performed in order to create a more unified neurocognitive model of congenital amusia.

Poor performance in face perception and memory in people with congenital amusia do not support the modularity of the mind hypothesis, which is the view that certain specialized domains of cognition are functionally distinct from each other. Although recent studies have suggested the domain specificity of congenital amusia, this is still disputed, and many researchers have suggested that the amusia might involve some visual processing deficits, such as face recognition and face memory. To that effect, some work has shown that amusia is not restricted to music, but is also related to language processing and spatial processing [24,66–68]. For confirmation purposes, further studies should explore whether congenital amusia extends to additional aspects of face processing and face memory.

The current results suggest a link between congenital amusia and deficits in processing of visual object (face perception and face memory). The results suggested that amusics’ deficits might not be restricted to musical domains. It is important to point out, that congenital amusia might be divided into several subtypes, such as vocal amusia, instrumental amnesia, musical agraphia, musical amnesia, disorders of rhythm, receptive amusia [69], expressive amusia [70] and musical anhedonia [71]. Prior studies have shown that expressive amusics have no problem in perceiving pitch, but are poor singers when singing familiar melodies [72] or when imitating unfamiliar pitch patterns [73]. Althought MBEA is currently widely used for the diagnosis of congenital amusia, some studies have shown that using the result of the first three subtests (scale, contour, and interval) to examine the performance of pitch processing is not sufficient to discern congenital amusia from the other aforementioned amusia subcategories. The present studies didn’t control the background in musical education and abilities of memory for both groups. It is not clear which categories our amusic participants belonged; a more discriminatory diagnostic instrument should be developed and more confound variables should be controlled. In broader issues, congenital amusia has been reported to correlate with general cognitive deficits [74–76]. Specifically, their results showed that music perception ability was associated with other cognitive abilities, such as attention, memory, or executive function, which may contribute to the appearance of overlapping processing deficits. Finally, the participants in the current study were mostly young women. Future studies should recruit an equal number of male and female participants to observe any potential sex differences existing for those with congenital amusia.

## Conclusion

In summary, the findings of this study suggest that there might be a new aspect of the congenital amusia impairment, i.e., a reduced face perception and face memory abilities in congenital amusics compared to a control group. In addition, the findings provide some tentative evidence that congenital amusia impairment might be extended to the visual processing domain.

## References

[1] Peretz I. The Biological Foundations of Music.; 2013 pp. 551–564.

[2] Royal I, Paquette S, Tranchant P. Musical Disorders.; 2019.

[3] Kalmus H, Fry DB. On tune deafness (dysmelodia): frequency, development, genetics and musical background. Ann Hum Genet. 1980; 43: 369–382. 10.1111/j.1469-1809.1980.tb01571.x 7396411

[4] Nan Y, Sun YN, Peretz I. Congenital amusia in speakers of a tone language: association with lexical tone agnosia. Brain. 2010; 133: 2635–2642. 10.1093/brain/awq178 20685803

[5] Peretz I, Vuvan DT. Prevalence of congenital amusia. Eur J Hum Genet. 2017; 25: 625–630. 10.1038/ejhg.2017.15 28224991 PMC5437896

[6] Ayotte J, Peretz I, Hyde K. Congenital amusia—A group study of adults afflicted with a music-specific disorder. Brain. 2002; 125: 238–251. 10.1093/brain/awf028 11844725

[7] Peretz I, Ayotte J, Zatorre RJ, Mehler J, Ahad P, Penhune VB et al Congenital amusia: A disorder of fine-grained pitch discrimination. Neuron. 2002; 33: 185–191. 10.1016/s0896-6273(01)00580-3 11804567

[8] Peretz I, Hyde KL. What is specific to music processing? Insights from congenital amusia. Trends Cogn Sci. 2003; 7: 362–367. 10.1016/s1364-6613(03)00150-5 12907232

[9] Thompson WF, Marin MM, Stewart L. Reduced sensitivity to emotional prosody in congenital amusia rekindles the musical protolanguage hypothesis. P Natl Acad Sci A. 2012; 109: 19027–19032. 10.1073/pnas.1210344109 PMC3503229 23112175

[10] Gosselin N, Paquette S, Peretz I. Sensitivity to musical emotions in congenital amusia. Cortex 2015; 71: 171–182. 10.1016/j.cortex.2015.06.022 26226563

[11] Stewart L. Fractionating the musical mind: insights from congenital amusia. Curr Opin Neurobiol. 2008; 18: 127–130. 10.1016/j.conb.2008.07.008 18694826

[12] Foxton JM, Dean JL, Gee R, Peretz I, Griffiths TD. Characterization of deficits in pitch perception underlying ‘tone deafness’. Brain. 2004; 127: 801–810. 10.1093/brain/awh105 14985262

[13] Hyde KL, Peretz I. Brains that are out of tune but in time. Psychol Sci 2004; 15: 356–360. 10.1111/j.0956-7976.2004.00683.x 15102148

[14] Stewart L, Walsh V. Congenital amusia: All the songs sound the same. Curr Biol. 2002; 12: 420–421. 10.1016/s0960-9822(02)00913-2 12123591

[15] Marin MM, Gingras B, Stewart L. Perception of musical timbre in congenital amusia: Categorization, discrimination and short-term memory. Neuropsychologia. 2012; 50: 367–378. 10.1016/j.neuropsychologia.2011.12.006 22201556

[16] Tillmann B, Schulze K, Foxton JM. Congenital amusia: A short-term memory deficit for non-verbal, but not verbal sounds. Brain Cognition. 2009; 71: 259–264. 10.1016/j.bandc.2009.08.003 19762140

[17] Patel AD, Wong M, Foxton J, Lochy A, Peretz I. Speech intonation perception deficits in musical tone deafness (congenital amusia). Music Percept. 2008; 25: 357–368.

[18] Nguyen S, Tillmann B, Gosselin N, Peretz I. Tonal Language Processing in Congenital Amusia.Ann N Y Acad Sci. 2009; 1169: 490–493. 10.1111/j.1749-6632.2009.04855.x 19673828

[19] Liu F, Patel AD, Fourcin A, Stewart L. Intonation processing in congenital amusia: discrimination, identification and imitation. Brian. 2010; 133: 1682–1693. 10.1093/brain/awq089 20418275

[20] Jiang CM, Hammn J, Lim V, Kirk I, Yang YF. Processing melodic contour and speech intonation in congenital amusics with Mandarin Chinese. Neuropsychologia. 2010; 48: 2630–2639. 10.1016/j.neuropsychologia.2010.05.009 20471406

[21] Jiang C, Hamm JP, Lim VK, Kirk IJ, Chen X, Yang Y. Amusia results in abnormal brain activity following inappropriate intonation during speech comprehension. Plos One. 2012; 7: e41411 10.1371/journal.pone.0041411 22859982 PMC3407197

[22] Perrachione TK, Fedorenko EG, Vinke L, Gibson E, Dilley LC. Evidence for Shared Cognitive Processing of Pitch in Music and Language. Plos One. 2013; 8. 10.1371/journal.pone.0073372 PMC3744486 23977386

[23] Fiveash A, Pammer K. Music and language: Do they draw on similar syntactic working memory resources? Psychol Music. 2014; 42: 190–209.

[24] Douglas KM, Bilkey DK. Amusia is associated with deficits in spatial processing. Nat Neurosci 2007; 10: 915–921. 10.1038/nn1925 17589505

[25] Tao W, Huang H, Li H, Lu Y, Tao X. Spatial representation of pitch in congenital amusia (In Chinese). Psychol Sci. 2015; 3: 733–738.

[26] Lu XJ, Sun YN, Ho HT, Thompson WF. Pitch contour impairment in congenital amusia: New insights from the Self-paced Audio-visual Contour Task (SACT). Plos One. 2017; 12. 10.1371/journal.pone.0179252 PMC5472285 28617864

[27] Sun YA, Lu XJ, Ho HT, Thompson WF. Pitch discrimination associated with phonological awareness: Evidence from congenital amusia. Sci Rep. 2017; 7. 10.1038/srep44285 PMC5347159 28287166

[28] Tillmann B, Jolicoeur P, Ishihara M, Gosselin N, Bertrand O, Rossetti Y et al The Amusic Brain: Lost in Music, but Not in Space. Plos One. 2010; 5. 10.1371/journal.pone.0010173 PMC2858073 20422050

[29] Williamson VJ, Cocchini G, Stewart L. The relationship between pitch and space in congenital amusia. Brain Cognition. 2011; 76: 70–76. 10.1016/j.bandc.2011.02.016 21440971

[30] Paquette S, Li HC, Corrow SL, Buss SS, Barton J, Schlaug G. Developmental Perceptual Impairments: Cases When Tone-Deafness and Prosopagnosia Co-occur. Front Hum Neuroscience. 2018; 12. 10.3389/fnhum.2018.00438 PMC6218620 30425629

[31] Corrow SL, Stubbs JL, Schlaug G, Buss S, Paquette S, Duchaine B et al Perception of musical pitch in developmental prosopagnosia. Neuropsychologia 2019; 124: 87–97. 10.1016/j.neuropsychologia.2018.12.022 30625291 PMC10262916

[32] Couvignou M, Peretz I, Ramus F. Comorbidity and cognitive overlap between developmental dyslexia and congenital amusia. Cogn Neuropsychol 2019; 36: 1–17. 10.1080/02643294.2019.1578205 30785364

[33] Trehub SE. Musical predispositions in infancy. Ann Ny Acad Sci. 2001: 1–16. 10.1111/j.1749-6632.2001.tb05721.x 11458822

[34] Kim J, Levitin DJ. Configural processing in melody recognition. *Canadian Acoustics* 2002; 3: 156–157.

[35] EO A. How many music centers are in the brain?. Ann N Y Acad Sci 2001; 930: 273–280. 10.1111/j.1749-6632.2001.tb05738.x 11458834

[36] Tanaka JW, Farah MJ. Parts and wholes in face recognition. The Quarterly Journal of Experimental Psychology, 46(2), 225–245. 10.1080/14640749308401045 8316637

[37] Ellis AW, Burton AM, Young A, Flude BM. Repetition priming between parts and wholes: Tests of a computational model of familiar face recognition. Brit J Psychol. 1997; 88: 579–608.

[38] Farah MJ, Wilson KD, Drain M, Tanaka JN. What is "special" about face perception? Psychol Rev 1998; 105: 482–498. 10.1037/0033-295x.105.3.482 9697428

[39] Goffaux V, Rossion B. Faces are "spatial"—Holistic face perception is supported by low spatial frequencies. J Exp Psychol Human. 2006; 32: 1023–1039. 10.1037/0096-1523.32.4.1023 16846295

[40] McKone E, Kanwisher N, Duchaine BC. Can generic expertise explain special processing for faces? Trends Cogn Sci. 2007; 11: 8–15. 10.1016/j.tics.2006.11.002 17129746

[41] Freiwald WA, Tsao DY, Livingstone MS. A face feature space in the macaque temporal lobe. Nat Neurosci. 2009; 12: 1187–1196. 10.1038/nn.2363 19668199 PMC2819705

[42] Hyde KL, Zatorre RJ, Peretz I. Functional MRI Evidence of an Abnormal Neural Network for Pitch Processing in Congenital Amusia. Cereb Cortex. 2011; 21: 292–299. 10.1093/cercor/bhq094 20494966

[43] Stewart L. Characterizing congenital amusia. *Q J Exp Psychol* 2011; 64: 625–638. 10.1080/17470218.2011.552730 21409740

[44] Avidan G, Behrmann M. Functional MRI reveals compromised neural integrity of the face processing network in congenital prosopagnosia. Curr Biol. 2009; 19: 1146–1150. 10.1016/j.cub.2009.04.060 19481456 PMC2711224

[45] Peretz I. Neurobiology of Congenital Amusia. Trends Cogn Sci. 2016; 20: 857–867. 10.1016/j.tics.2016.09.002 27692992

[46] Tillmann B, Leveque Y, Fornoni L, Albouy P, Caclin A. Impaired short-term memory for pitch in congenital amusia. Brain Res. 2016; 1640: 251–263. 10.1016/j.brainres.2015.10.035 26505915

[47] Caclin A, Tillmann B. Musical and verbal short-term memory: insights from neurodevelopmental and neurological disorders.Ann NY Acad Sci. 2018; 1423: 155–165. 10.1111/nyas.13733 29744897

[48] Gosselin N, Jolicoeur P, Peretz I. Impaired Memory for Pitch in Congenital Amusia.; 2009 pp. 270–272. 10.1111/j.1749-6632.2009.04762.x 19673791

[49] Peretz I, Champod AS, Hyde K. Varieties of musical disorders—The Montreal battery of evaluation of amusia.; 2003 pp. 58–75. 10.1196/annals.1284.006 14681118

[50] Bai L, Ma H, Huang YX, Luo YJ. The development of native Chinese affective picture system-A pretest in 46 college students(in Chinese). Chinese mental health journal 2005; 19: 719–722.

[51] Yin RK. Looking at upside-down faces. *Journal of experimental Psychology* 1969: 141–145.

[52] Bartlett JC, Searcy J. Inversion and configuration of faces. Cogn Psychol 1993; 25: 281–316. 10.1006/cogp.1993.1007 8354050

[53] Van Belle G, De Smet M, De Graef P, Van Gool L, Verfaillie K. Configural and featural processing during face perception: A new stimulus set. Behav Res Methods. 2009; 41: 279–283. 10.3758/BRM.41.2.279 19363168

[54] Gauthier I, Skudlarski P, Gore JC, Anderson AW. Expertise for cars and birds recruits brain areas involved in face recognition. Nat Neurosci. 2000; 3: 191–197. 10.1038/72140 10649576

[55] Maurer D, Le Grand R, Mondloch CJ. The many faces of configural processing. Trends Cogn Sci. 2002; 6: 255–260 10.1016/s1364-6613(02)01903-4 12039607

[56] Bedard C, Belin P. A "voice inversion effect?". Brain Cognition. 2004; 55: 247–249. 10.1016/j.bandc.2004.02.008 15177788

[57] Leveque Y, Fauvel B, Groussard M, Caclin A, Albouy P, Platel H et al Altered intrinsic connectivity of the auditory cortex in congenital amusia. *J Neurophysiol* 2016; 116: 88–97. 10.1152/jn.00663.2015 27009161 PMC4961744

[58] Peretz I, Zatorre RJ. Brain organization for music processing. Annu Rev Psychol. 2005; 56: 89–114. 10.1146/annurev.psych.56.091103.070225 15709930

[59] Satoh M, Takeda K, Murakami Y, Onouchi K, Inoue K, Kuzuhara S. A case of amusia caused by the infarction of anterior portion of bilateral temporal lobes. Cortex. 2005; 41: 77–83. 10.1016/s0010-9452(08)70180-1 15633709

[60] Nan Y, Friederici AD. Differential roles of right temporal cortex and broca's area in pitch processing: Evidence from music and mandarin. Hum Brain Mapp. 2013; 34: 2045–2054. 10.1002/hbm.22046 22431306 PMC6870388

[61] Sergent J, Ohta S, Macdonald B.Functional neuroanatomy of face and object processing a positron emission tomography study. Brain.1992; 115: 15–36. 10.1093/brain/115.1.15 1559150

[62] Damasio H, Grabowski TJ, Tranel D, Hichwa RD, Damasio AR. A neural basis for lexical retrieval. Nature. 1996; 380: 499–505. 10.1038/380499a0 8606767

[63] Moore CJ, Price CJ. A functional neuroimaging study of the variables that generate category-specific object processing differences. Brain. 1999; 122: 943–962. 10.1093/brain/122.5.943 10355678

[64] Malach R, Reppas JB, Benson RR, Kwong KK, Jiang H, Kennedy WA et al Object-related activity revealed by functional magnetic resonance imaging in human occipital cortex. Proc Natl Acad Sci U S A. 1995; 92: 8135–8139. 10.1073/pnas.92.18.8135 7667258 PMC41110

[65] DEsposito M, Zarahn E, Aguirre GK, Shin RK, Auerbach P, Detre JA. The effect of pacing of experimental stimuli on observed functional MRI activity. Neuroimage. 1997; 6: 113–121. 10.1006/nimg.1997.0281 9299385

[66] Curran CP, Williams MT, Vorhees CV, Patel KV, Nebert DW. Genetic susceptibility to PCB-induced developmental neurotoxicity. Birth Defects Res A. 2008; 82: 290.

[67] Williamson VJ, Stewart L. Memory for pitch in congenital amusia: Beyond a fine-grained pitch discrimination problem. Memory. 2010; 18: 657–669. 10.1080/09658211.2010.501339 20706954

[68] Tillmann B, Burnham D, Nguyen S, Grimault N, Gosselin N, Peretz I. Congenital Amusia (or Tone-Deafness) Interferes with Pitch Processing in Tone Languages. Front Psychol. 2011; 2: 120 10.3389/fpsyg.2011.00120 21734894 PMC3119887

[69] Berman IW. Musical functioning, speech lateralization and the amusias. S Afr Med J.1981; 59: 78–81. 7008213

[70] Goldstein AG, Stephenson B, Chance J. Face recognition memory: Distribution of false alarms. Bulletin of the psychonomic Society. 1977; 9: 416–418.

[71] Mas-Herrero E, Zatorre R, Rodriguez-Fornells A, Marco-Pallarés J. Dissociation between Musical and Monetary Reward Responses in Specific Musical Anhedonia. *Current biology*: *CB* 2014; 24. 10.1016/j.cub.2014.01.068 24613311

[72] Dalla Bella S, Giguère JF, And Peretz I. Singing proficiency in the general population. Journal Acoustic society of America 2007: 1182–1189. 10.1121/1.2427111 17348539

[73] Pfordresher PQ, Brown S. Poor-pitch singing in the absence of "tone deafness". Musci Percept. 2007; 25: 95–115.

[74] Sarkamo T, Tervaniemi M, Soinila S, Autti T, Silvennoinen HM, Laine M et al Amusia and Cognitive Deficits after Stroke Is There a Relationship? Ann N Y Acad Sci.2009;1169: 441–445. 10.1111/j.1749-6632.2009.04765.x 19673821

[75] Wen Y, Nie X, Wu D, Liu H, Zhang P, Lu X. Amusia and cognitive deficits in schizophrenia: Is there a relationship? Schizophr Res. 2014; 157: 60–62. 10.1016/j.schres.2014.05.029 24957355

[76] Hatada S, Sawada K, Akamatsu M, Doi E, Minese M, Yamashita M et al Impaired musical ability in people with schizophrenia. J Psychiatr Neurosci. 2014; 39: 118–126. 10.1503/jpn.120207 PMC3937280 24119791

